# Determination of Phase Transition and Critical Behavior of the As-Cast GdGeSi-(X) Type Alloys (Where X = Ni, Nd and Pr)

**DOI:** 10.3390/ma14010185

**Published:** 2021-01-04

**Authors:** Piotr Gębara, Mariusz Hasiak

**Affiliations:** 1 Department of Physics, Częstochowa University of Technology, Armii Krajowej 19, 42-200 Częstochowa, Poland; 2 Department of Mechanics, Materials and Biomedical Engineering, Wrocław University of Science and Technology, Smoluchowskiego 25, 50-370 Wrocław, Poland; mariusz.hasiak@pwr.edu.pl

**Keywords:** magnetocaloric effect, Gd-based alloys, phase transitions, critical behavior

## Abstract

The aim of the paper is to present a study of the magnetocaloric effect and the nature of phase transition in the Gd_80_Ge_15_Si_5_ (S1), Gd_75_Ge_15_Si_5_Ni_5_ (S2), Gd_75_Ge_15_Si_5_Pr_5_ (S3) and Gd_75_Ge_15_Si_5_Nd_5_ (S4) alloys. The magnetic entropy changes determined for studied samples, under external magnetic field ~3T, were 11.91, 12.11, 5.08 and 4.71 J/(kg K) for S1, S2, S3 and S4, respectively. The values of refrigerant capacity (under ~3T) were 164, 140, 160 and 140 J/kg for S1, S2, S3 and S4, respectively. The first order phase transition was detected for samples S1 and S2, while specimens S3 and S4 manifested the second order phase transition at the Curie point (*T_C_*). The analysis of temperature evolution of the exponent *n* (ΔS_M_ = C·(B_max_)^n^) showed the validity of this method in detecting either the first or the second order phase transition and the structural transition. The analysis of critical behavior was carried out for samples S3 and S4. The critical exponents and precise *T_C_* values were calculated. The ascertained critical exponents were used to determine the theoretical value of the exponent *n*, which corresponded well with experimental result.

## 1. Introduction

Magnetic materials revealing the magnetocaloric effect (MCE) have been intensively studied for over 20 years. The great interest in magnetocaloric materials (MCMs) was started in 1997 after the discovery of a giant MCE in the Gd_5_Si_2_Ge_2_ alloy by Pecharsky and Gschneidner Jr. [1]. The Gd_5_Ge_2_Si_2_ alloy and pure Gd showed a trend of development of MCMs. For more than two decades, many MCMs were produced, such as La(Fe,Si)_13_-type alloys [2,3], manganites [4,5], Heusler alloys [6,7,8] and many others. Magnetocaloric properties of mentioned alloys are characterized by the first or the second order phase transition (FOPT, SOPT). MCMs manifesting FOPT have a higher degree of both the magnetic entropy change ΔS_M_ and the adiabatic temperature change ΔT_ad_, related to the former value, than materials with SOPT. However, the main advantage of materials with SOPT is the working temperature range. Law and coworkers [9] were of the opinion that an ideal MCM should lay at the borderline between FOPT and SOPT materials. They presented an original approach to the determination of the phase transition based on magnetocaloric data. Hasiak [10] presented results of magnetocaloric measurements for the as-cast GdSiGe alloys modified with Ni and Ce. In our previous work [11], the critical behavior of the GdSiGeCe alloy was studied and the values of critical exponents for the Ce-doped alloy were found to be *β* = 0.376, *γ* = 1.032 and *δ* = 3.385. They were determined using the Kouvel–Fisher technique [12].

The present paper aims to bring forward a study of the character of transition in the GdGeSi-X-type alloys (where X = Pr, Nd, Ni) based on temperature dependences of the exponent *n* (ΔS_Mmax_ = C(B_max_)^n^). Moreover, for samples revealing SOPT, the Kouvel–Fisher analysis was applied in order to determine critical exponents, and a theoretical value of the exponent *n* was calculated.

## 2. Materials and Methods

Samples of nominal composition Gd_80_Ge_15_Si_5_, Gd_75_Ge_15_Si_5_Ni_5_, Gd_75_Ge_15_Si_5_Pr_5_ and Gd_75_Ge_15_Si_5_Nd_5_ (wt.%) were prepared by arc-melting of high purity constituent elements under low pressure of the Ar atmosphere. Samples in the form of tablets with a mass of about 10 g were remelted ten times in order to ensure their homogeneity. In order to compensate evaporation of Gd, an excess amount of 5wt.% of the element was applied. The chemical composition of the produced materials was checked by a scanning electron microscope (SEM, Quanta 250, FEI, Hillsboro, OR, USA) equipped with an energy dispersive X-ray spectroscopy (EDS) detector working in secondary electrons (SE) mode [10,13]. The structure was examined by X-ray diffraction using a Bruker D8 Advance diffractometer (Bruker AXS Gmbh, Karlsruhe, Baden-Wurtemberg, Germany) equipped with a Cu tube and a semiconductor LynxEye detector. According to EDS and X-ray examinations, the chemical composition of the studied samples is close to nominal, and the structure is single-phase. Magnetic measurements were carried out using a Quantum Design VersaLab (Quantum Design, San Diego, CA, USA) cryogen-free vibrating sample magnetometer working in a wide range of temperatures and in magnetic fields up to ~3T. An investigation of phase transition in GdGeSi-based alloys was performed by taking measurements of specific heat capacity versus temperature at zero external magnetic fields within a two-tau model with help of a Physical Properties Measurement System (PPMS) (Quantum Design).

The magnetocaloric effect was investigated indirectly on the basis of field dependences of magnetization recorded over a wide range of temperatures. In order to calculate ΔS_M_, the thermomagnetic Maxwell’s relation was used [14]:

(1) $$\Delta S_{M}(T,\Delta H)=\mu_{0}\int_{0}^{H}{\left(\frac{\partial M(T,H)}{\partial T}\right)}_{H}dH$$

where *T*, *μ*_0_, *H* and *M* are temperature, magnetic permeability, magnetic field strength and magnetization, respectively.

The magnetocaloric characterization of studied samples was supplemented by calculations of refrigeration capacity, according to the following relation [15]:

(2) $$RC(\delta T,H_{MAX})=\int_{T_{cold}}^{{}_{T_{hot}}}\Delta S_{M}(T,H_{MAX})dT,$$

where *RC* is refrigerant capacity, *δT* = *T_hot_* − *T_cold_* is the temperature range of the thermodynamic cycle (*δT* corresponds to the full width at half maximum of magnetic entropy change peak), and *H_MAX_* is the maximum value of the external magnetic field.

The analysis of phase transition was carried out using Arrott plots and the temperature dependence of the exponent *n*. The exponent *n* was found using Franco et al.’s phenomenological relation describing the field dependence of the magnetic entropy change written in the following form [16,17]:

(3) $$\Delta S_{M\max}=C\cdot {\left(B_{MAX}\right)}^{n},$$

where *C* is a proportionality constant depending on temperature, and *n* is the exponent related to the magnetic state of the material.

Świerczek [18] proposed a simple modification of the relation (3), which allows determining the exponent n in a direct way:

(4) $$\ln\Delta S_{M\max}=\ln C+n\ln(B_{\max}).$$

Linear regression of Equation (4) allowed finding out the exponent *n* directly from the slope of a straight line. The correlation coefficient ascertained during the present studies was 0.998 or higher.

The critical exponents for samples doped by Pr and Nd were determined using the Kouvel–Fisher technique [19].

## 3. Results and Discussions

**Figure 1:**
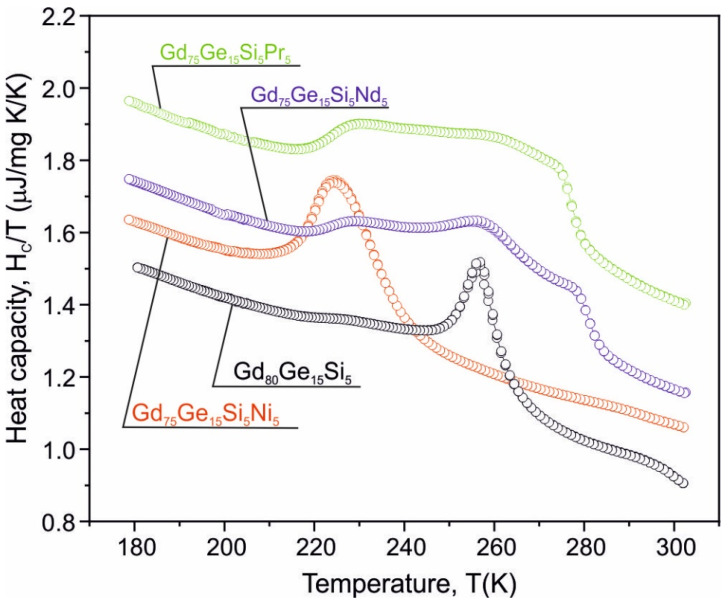
The heat capacity vs. temperature measured for the Gd_80_Ge_15_Si_5_, Gd_75_Ge_15_Si_5_Ni_5_ [10], Gd_75_Ge_15_Si_5_Pr_5_ [13] and Gd_75_Ge_15_Si_5_Nd_5_ alloys at zero external magnetic field.

The temperature dependence of heat capacity for the as-cast Gd_80_Ge_15_Si_5_, Gd_75_Ge_15_Si_5_Ni_5_, Gd_75_Ge_15_Si_5_Pr_5_ and Gd_75_Ge_15_Si_5_Nd_5_ (wt.%) alloys measured without an external magnetic field is presented in Figure 1. The Gd_80_Ge_15_Si_5_ and Gd_75_Ge_15_Si_5_Ni_5_ samples show the well-seen λ shape structural transition corresponding to the first order phase transition (FOPT) with the maxima at either 256 K or 225 K, respectively. For the Pr- and Nd-containing samples, the wide maxima within the temperature range of 220–280 K were observed. Decomposition of these curves within the mentioned temperature range leads to the distinction of three components with their maxima at different temperature values. This behavior seems to be related to the multiphase structure of the Gd_75_Ge_15_Si_5_Pr_5_ and Gd_75_Ge_15_Si_5_Nd_5_ alloys.

**Figure 2:**
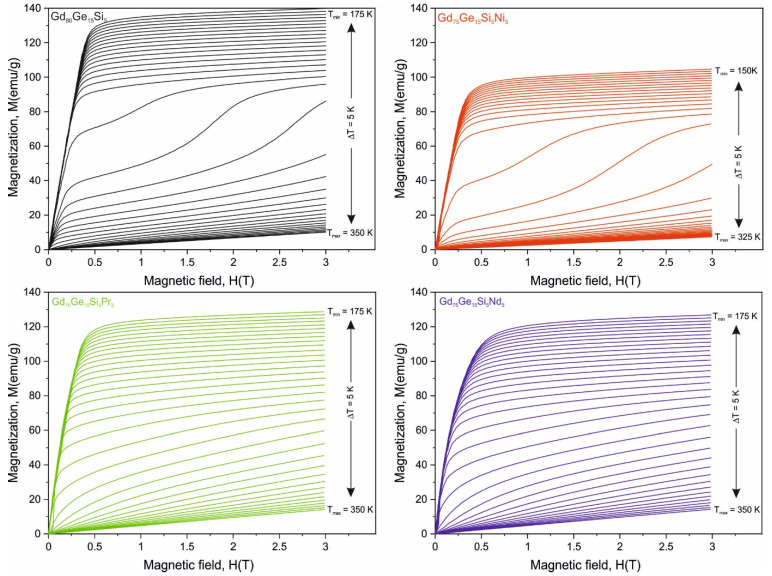
The set of isothermal magnetization characteristics for Gd_80_Ge_15_Si_5_, Gd_75_Ge_15_Si_5_Ni_5_ [10], Gd_75_Ge_15_Si_5_Pr_5_ and Gd_75_Ge_15_Si_5_Nd_5_ alloys measured in the vicinity of the Curie point with the step of *T* = 5 K for maximum external magnetic field up to 3 (the same scale on all X and Y axes was used to show the difference in magnetization between the investigated samples).

The isothermal magnetization curves *M*(*H*) recorded for all investigated alloys in the vicinity of the Curie temperature (*T_C_*) specific for each alloy (within the temperature range *T_C_* ± 50 K) with the step of 5 K for external magnetic fields up to 3 T are shown in Figure 2. All these *M*(*H*) curves show typical ferromagnetic character below *T_C_*, whereas only the linear dependence on temperature, distinctive for the paramagnetic state, was observed above the Curie point. It can be easily seen, however, that the Gd_80_Ge_15_Si_5_ and Gd_75_Ge_15_Si_5_Ni_5_ samples show different *M*(*H*) behavior in the vicinity of the Curie point than the samples with the addition of Pr and Ni. The surface area between two adjacent curves recorded with the step of *T* = 5 K for either the Gd_80_Ge_15_Si_5_ or the Gd_75_Ge_15_Si_5_Ni_5_ alloy near *T_C_* is several times larger than the corresponding one obtained for the samples containing either Pr or Nd. This behavior is strictly related to the occurrence of the first order phase transition in the formerly mentioned pair of alloys, which is also confirmed in Figure 1. The *M*(*H*) data together with results presented in Figure 1 clearly suggest that the Gd_75_Ge_15_Si_5_Pr_5_ and Gd_75_Ge_15_Si_5_Nd_5_ alloys close to Curie point show the second order phase transition.

**Figure 3:**
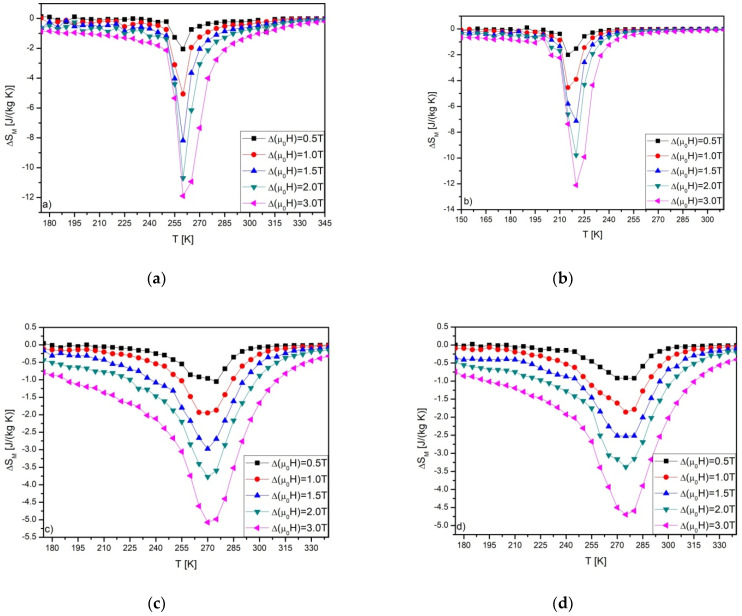
The ΔS_M_ vs. T curves calculated for: Gd_80_Ge_15_Si_5_ (**a**), Gd_75_Ge_15_Si_5_Ni_5_ (**b**) [10], Gd_75_Ge_15_Si_5_Pr_5_ (**c**) and Gd_75_Ge_15_Si_5_Nd_5_ (**d**) alloys.

**Figure 4:**
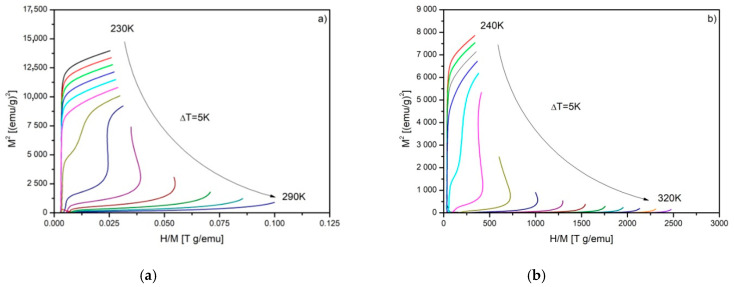

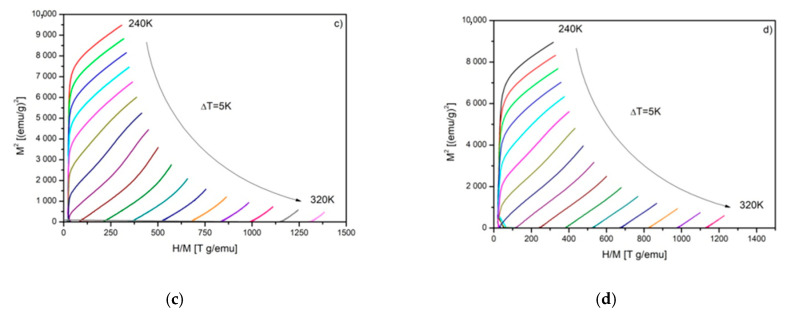
The M^2^ vs. (H/M) isotherms (Arrott plots) constructed for Gd_80_Ge_15_Si_5_ (**a**), Gd_75_Ge_15_Si_5_Ni_5_ (**b**), Gd_75_Ge_15_Si_5_Pr_5_ (**c**) and Gd_75_Ge_15_Si_5_Nd_5_ (**d**) alloys.

The ΔS_M_ vs. T curves calculated for Gd_80_Ge_15_Si_5_, Gd_75_Ge_15_Si_5_Ni_5_, Gd_75_Ge_15_Si_5_Pr_5_ and Gd_75_Ge_15_Si_5_Nd_5_ are depicted in Figure 3. The ΔS_M_ curves obtained for Gd_80_Ge_15_Si_5_ and Gd_75_Ge_15_Si_5_Ni_5_ alloys were presented previously elsewhere [10]; however, they are mentioned here once more for the purpose of further analysis. The highest values of ΔS_M_ were achieved for Gd_80_Ge_15_Si_5_ and Gd_75_Ge_15_Si_5_Ni_5_ alloys, and they were equal to 11.91 and 12.11 J/kg·K, respectively. The characteristic asymmetric shape, typical for materials with FOPT, can be noticed in Figure 3a,b. The “caret” shape was detected in the case of other samples, being a marker of SOPT. The values of ΔS_M_ and RC are collected in Table 1. It is clearly seen that the values of the RC revealed for all studied materials are almost the same. In order to start the analysis of the nature of phase transitions, the Arrott plots were constructed for all specimens (Figure 4).

The Banerjee criterion [19] of the nature of phase transition based on the slope of Arrott plots provides the preliminary interpretation of results. The Gd_80_Ge_15_Si_5_ and Gd_75_Ge_15_Si_5_Ni_5_ alloy samples manifest the FOPT in the vicinity of the Curie temperature *T_C_*, due to the characteristic “s-shape” of the Arrott plots in that temperature region. In the case of samples doped with Pr and Nd, a monotonic increase is observed. The positive slope of the Arrott plots depicted in Figure 4c,d suggests an occurrence of the SOPT in these samples.

**Figure 5:**
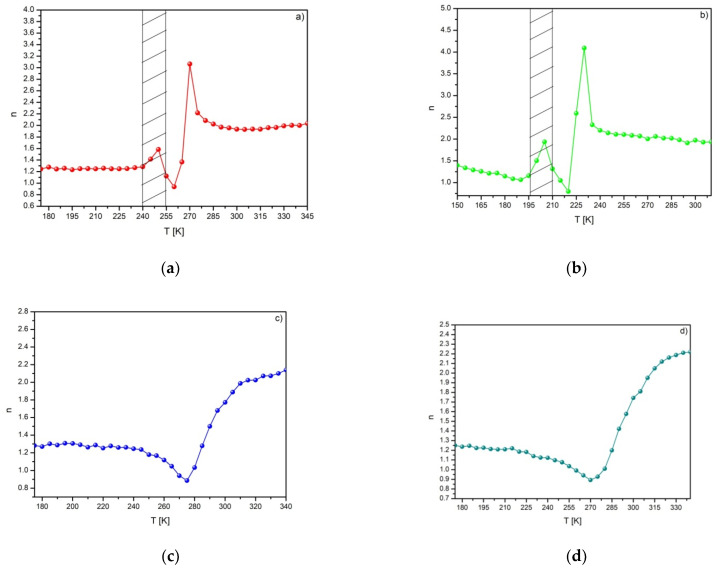
The *n* vs. T curves found for Gd_80_Ge_15_Si_5_ (**a**), Gd_75_Ge_15_Si_5_Ni_5_ (**b**), Gd_75_Ge_15_Si_5_Pr_5_ (**c**) and Gd_75_Ge_15_Si_5_Nd_5_ (**d**) alloys.

Further analysis based on the temperature dependence of the exponent *n* was determined from Equation (3). The *n* vs. T curves are shown in Figure 5. As was shown in [16,17], the value of the exponent *n* is strongly dependent on the magnetic state of the sample. Provided that materials obey the Curie–Weiss law, the exponent *n* takes the value of either 1 or 2 for either the ferro- or the paramagnetic state, respectively. At the Curie point, however, the exponent *n* is related with values of critical exponents, according to the following relation [16,17]:

(5) $$n=1+\frac{1}{\delta \left(1-\frac{1}{\beta }\right)}$$

where *β* and *δ* are critical exponents.

Taking into account the critical exponents delivered by Landau mean field theory (*β* = 0.5, *γ* = 1, *δ* = 3) and the Relation (5), *n* equals to 2/3. It is worth remembering that the mean field theory describes materials with SOPT. However, Law and coworkers showed different behavior of the temperature evolution of the exponent *n* [9]. They demonstrated, on the basis of the Bean–Rodbell model, that it is possible to identify not only the SOPT, but also the FOPT and the structural transformation. They reported the characteristic peak just before the Curie point for the Ni-Mn-In-Co Heusler alloy and related it to the martensitic-austenitic transition. The temperature dependences of the exponent *n* found for the Gd_80_Ge_15_Si_5_ and the Gd_75_Ge_15_Si_5_Ni_5_ alloys reveal the characteristic jump of the exponent *n* in the vicinity of the *T_C_* and the similar characteristic peak before the *T_C_*. It can be related to the transformation from monoclinic to orthorhombic structure induced in the GdGeSi-type alloys by the magnetic field [20].

The observed peak values of the exponent *n* (marked by the dashed area in Figure 5a,b) correspond to the structural transition. The temperature behavior of exponent *n* in the case of specimens modified by Pr and Nd is typical for materials with SOPT (Figure 5c,d). The values of the exponent calculated in the vicinity of the *T_C_* are 0.8863 and 0.8933 for Gd_80_Ge_15_Pr_5_ and Gd_75_Ge_15_Si_5_Nd_5_ alloys, respectively.

The confirmation of the occurrence of the SOPT in both the Gd_75_Ge_15_Si_5_Pr_5_ and the Gd_75_Ge_15_Si_5_Nd_5_ alloy samples allowed conducting research on the critical phenomena in the region of magnetic phase transition. The Arrott plots presented in Figure 4c,d are almost straight lines. These data (Arrott plots) and linear regression were used for the determination of both the spontaneous magnetization *M_S_* and the inverse susceptibility *1*/*χ*.

The SOPT is described by a system of critical exponents. The evolution of spontaneous magnetization *M_S_*, inverse susceptibility *1*/*χ* and isothermal magnetization at *T_C_* are strongly related to critical exponents *β*, *γ* and *δ*, respectively. These relations in the mathematical form are as follows [21]:

(6) $$M_{S}(T)=M_{0}{\left(-\varepsilon \right)}^{\beta },\;\varepsilon <0,\;T<T_{C},$$

(7) $$\chi_{0}{\left(T\right)}^{-1}=\left(\frac{H_{0}}{M_{0}}\right)\varepsilon^{\gamma },\;\varepsilon >0,\;T>T_{C},$$

(8) $$M=DH^{\frac{1}{\delta }},\;\varepsilon =0,\;T=T_{C},$$

where *ε* = (*T* − *T_C_*)/*T_C_* means the reduced temperature, *M*_0_, *H*_0_ and *D* are critical amplitudes, *H* is the applied field and *M* is magnetization.

**Figure 6:**
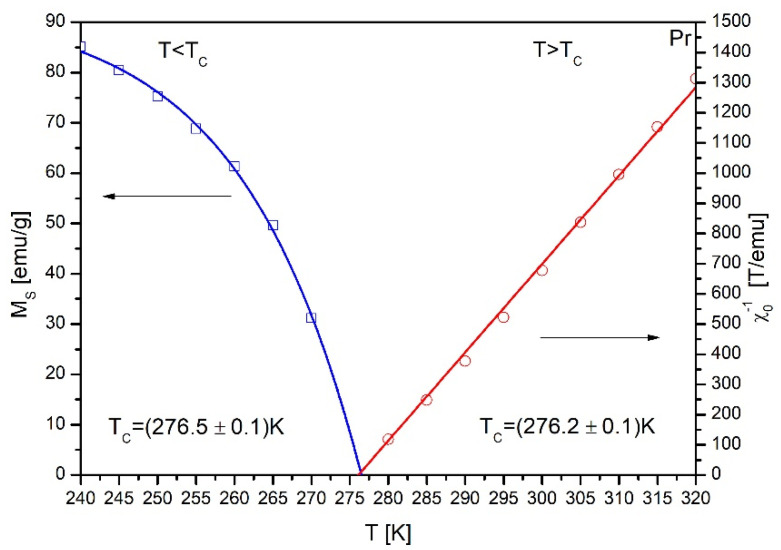
The temperature dependences of the spontaneous magnetization *M_S_* and the inverse initial susceptibility *χ*_0_^−1^ of the as-cast Gd_75_Ge_15_Si_5_Pr_5_ alloy.

The linear extrapolation of the M^2^ vs. *1*/*χ* isotherms allowed determining both the spontaneous magnetization *M_S_* and the inverse initial susceptibility *1*/*χ*. The temperature dependences of *M_S_* and *1*/*χ* are shown in Figure 6. These plots allowed us to determine a more precise value of the Curie temperature, which equals to either 276.5 or 276.2K for either *T* < *T_C_* or *T* > *T_C_* range, respectively.

Kouvel and Fisher [12] proposed a method for calculation of the critical exponents based on a simple modification of Relations (6) and (7). According to the Kouvel–Fisher technique, these equations were rewritten in the following form:

(9) $$\frac{M_{S}(T)}{\frac{dM_{S}(T)}{dT}}=\frac{T-T_{C}}{\beta },$$

(10) $$\frac{\chi_{0}^{-1}(T)}{\frac{d\chi_{0}^{-1}(T)}{dT}}=\frac{T-T_{C}}{\gamma }.$$

**Figure 7:**
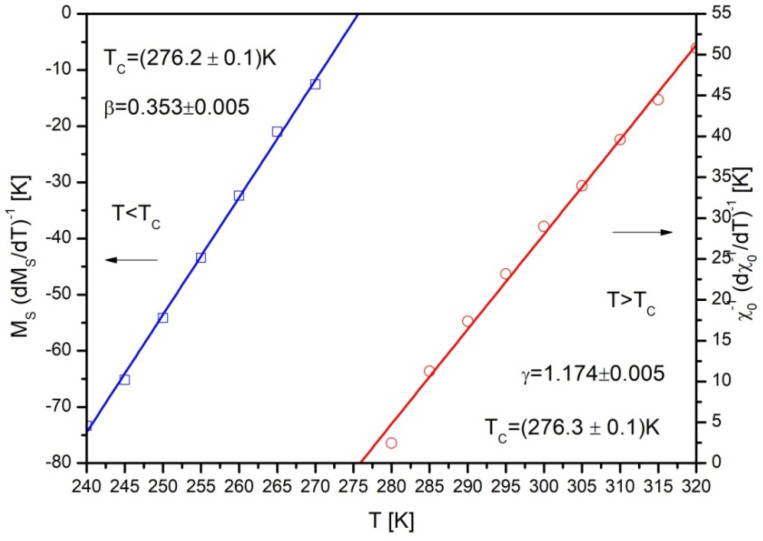
The Kouvel–Fisher plots for determination of *β* and *γ* in the Gd_75_Ge_15_Si_5_Pr_5_ alloy.

Such simple linearization with slopes 1/*β* and 1/*γ* allowed revealING values *β* and *γ* by linear fitting of Kouvel–Fisher plots (Figure 7). Moreover, these plots delivered the most precise information about the Curie temperature.

The values of critical exponents *β* and *γ* determined by Kouvel-Fisher plots were equal to 0.353 and 1.174, respectively. Such results correspond well with critical exponents delivered by other researchers [11,22,23].

The last critical exponent *δ* was calculated from the Widom scaling relation [24]:

(11) $$\delta =1+\frac{\gamma }{\beta }.$$

Inserting the already calculated values of *β* and *γ* to Equation (11), one can find *δ* to be 4.326. Independently, the value of *δ* was also determined using Equation (8) rewritten in the following form:

(12) $$\ln M=\ln D+\frac{1}{\delta }\ln H.$$

**Figure 8:**
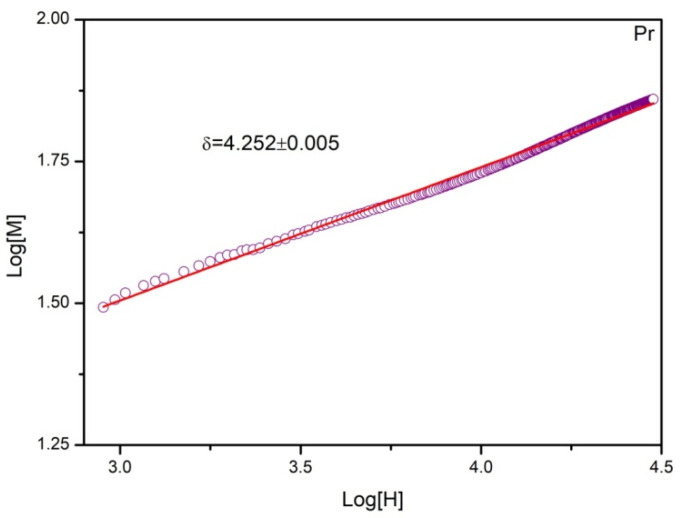
The field dependence *H* of magnetization *M* on a log–log scale recorded at 275 K for the as-quenched Gd_75_Ge_15_Si_5_Pr_5_ alloy. The blue line is the best linear fit according to Equation (12).

The field dependence of magnetization (*M* vs. *H*) in the vicinity of *T_C_* in the log–log scale is depicted in Figure 8. Since the Curie temperature determined by Kouvel–Fisher plots was found to be 276.2 K, the curve recorded at 275 K was selected for analysis as the closest approximation. The linear fitting delivered information about *δ* being equal to 4.252. Such a value corresponds well with the one calculated from the Widom relation.

**Figure 9:**
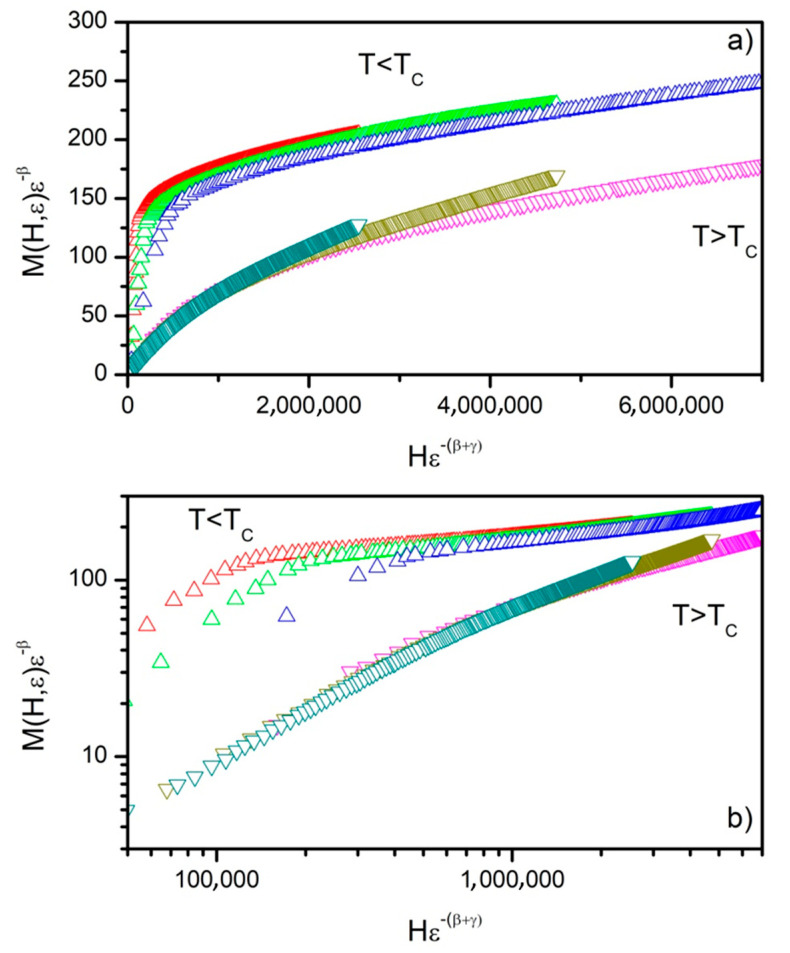
Scaling plots calculated for the Gd_75_Ge_15_Si_5_Pr_5_ alloy in as-cast state in a linear scale (**a**) and in a log–log scale (**b**).

The validation of calculated critical exponents (*β*, *γ* and *δ*) was performed using the magnetic equation of state [25]:

(13) $$M(H,\varepsilon )=\varepsilon^{\beta }f_{\pm }\left(\frac{H}{\varepsilon^{\beta +\gamma }}\right),$$

where *f*_±_ are regular functions, *f*_+_ being the one valid for the paramagnetic region (*T* > *T_C_*), while *f*_−_ holds for the ferromagnetic one (*T* < *T_C_*). Formula (13) expresses the dependence between *M*(*H*,*ε*) *ε^−β^* and *H ε*^−(*β* + *γ*)^ in the form of two curves. One of them is observed for temperature values lower than *T_C_* (ferromagnetic state) and the second one for temperature values higher than *T_C_* (paramagnetic state). According to Equation (13), the *M* vs. *H* data should collapse into two independent universal curves. The calculated critical exponents were used to construct the M vs. H plots depicted in Figure 9a. It can be noticed that the curves recorded for temperature values beneath the Curie point actually collapse into one universal curve, while the ones achieved for temperature values exceeding the *T_C_* collapse into the second one. The same data are depicted in the log–log scale in Figure 9b.

Such behavior confirms the validity of the determined critical exponents and proves that the Curie point is dependable. The exponents are in good agreement with the scaling hypothesis. The same procedure was applied for data achieved for the Gd_75_Ge_15_Si_5_Nd_5_ alloy. The values of the calculated critical exponents are collected in Table 2.

Taking into account Relation (5) and the determined critical exponents, the theoretical value of the exponent *n* for the Gd_75_Ge_15_Si_5_Pr_5_ alloy was calculated. This theoretical value was determined in two ways: first on the basis of the experimentally found value of *δ* and then from the value delivered by Widom scaling relation, and it was found to be either 0.8716 or 0.8739, respectively. In the case of sample doped with Nd, the theoretical values of the exponent *n* are also collected in Table 2, along with the critical exponents reported by other authors, which were used to determine the theoretical value of the exponent *n*.

The critical exponent *γ* determined for the Gd_75_Ge_15_Si_5_Pr_5_ and Gd_75_Ge_15_Si_5_Nd_5_ alloys corresponds well with the mean field theory. In the case of *β*, its value is closer to *β* from the 3D-Heisenberg model. As in the case of results published previously for the Gd_75_Ge_15_Si_5_Ce_5_ alloy [11], it is difficult to distinguish which model correctly describes magnetism in the produced alloys. The value of the exponent *n* found for samples modified with either Pr or Nd is in agreement with values delivered by analysis of magnetocaloric data.

## 4. Conclusions

Investigations on the magnetocaloric effect and the nature of phase transition in the Gd_80_Ge_15_Si_5_, Gd_75_Ge_15_Si_5_Ni_5_, Gd_75_Ge_15_Si_5_Pr_5_ and Gd_75_Ge_15_Si_5_Nd_5_ alloys were described in the present paper. The highest magnetic entropy change of similar value was measured for the Gd_80_Ge_15_Si_5_ and Gd_75_Ge_15_Si_5_Ni_5_ alloys. Further, the occurrence of either the FOPT or the SOPT was detected in the investigated materials by means of heat capacity measurements, the Arrott plots and the techniques employing temperature dependences of the exponent *n*. The characteristic peaks in the *n* vs. T curves (constructed for the Gd_80_Ge_15_Si_5_ and the Gd_75_Ge_15_Si_5_Ni_5_ alloys) correspond with the structural transitions occurring in the vicinity of the Curie temperature. In the case of samples doped with Pr and Nd, the same techniques confirmed the occurrence of SOPT at the *T_C_*. These results were confirmed by investigations on temperature dependence of the heat capacity of the examined alloys. Then, the critical behavior in the vicinity of *T_C_* was studied, resulting in the precise values of the Curie point for each alloy and the values of critical exponents. Subsequently, the theoretical value of the exponent *n* was determined on the basis of calculated critical exponents. It was found to correspond well with the experiments.

## Figures and Tables

**Table 1:** The values of ΔS_M_ and RC calculated for the investigated alloys at selected values of magnetic field.

Alloy	μ_0_H [T]	-S_M_ [J/(kg K)]	RC [J/kg]
Gd_80_Ge_15_Si_5_	0.5	2.05	16
	1	5.06	38
	1.5	8.18	60
	2	10.71	102
	3	11.91	164
Gd_75_Ge_15_Si_5_Ni_5_	0.5	2.00	20
	1	4.54	38
	1.5	7.13	66
	2	9.78	84
	3	12.11	140
Gd_75_Ge_15_Si_5_Pr_5_	0.5	1.05	22
	1	1.95	42
	1.5	2.98	72
	2	3.77	103
	3	5.08	160
Gd_75_Ge_15_Si_5_Nd_5_	0.5	0.92	21
	1	1.86	52
	1.5	2.53	76
	2	3.38	108
	3	4.71	140

**Table 2:** Critical exponents, the Curie temperature and the exponent *n* calculated for the Gd_75_Ge_15_Si_5_Pr_5_ and Gd_75_Ge_15_Si_5_Nd_5_ alloys together with values delivered by theoretical models. Abbreviations W and TW mean “Widom scaling relation” and “this work”, respectively.

Alloy	Ref.	*β*	*γ*	*δ*	*T_C_* [K]	n_exp_	n_theor_	n_W_
Gd_75_Ge_15_Si_5_Pr_5_ = Gd_4.8_Pr_0.4_Ge_2.0_Si_1.8_	TW	0.353	1.174	4.252 4.326 (W)	276.2 ± 0.1	0.8863	0.8716	0.8739
Gd_75_Ge_15_Si_5_Nd_5_ = Gd_4.8_Nd_0.4_Ge_2.0_Si_1.8_	TW	0.324	1.119	4.167 4.456 (W)	277.6 ± 0.1	0.8933	0.8849	0.8924
Gd_75_Ge_15_Si_5_Ce_5_ = Gd_4.8_Ce_0.4_Si_2.0_Ge_1.8_	[11]	0.376	1.032	3.385 3.745 (W)	275.8	-	0.822	0.8214
pure Gd	[22]	0.381	1.196	4.139	296	-	0.8513	-
Gd_5_Si_2_Ge_1.9_Cu_0.1_	[23]	0.38	1.15	4.03	-	-	0.8479	-
Gd_5_Si_2_Ge_1.9_Mn_0.1_	[23]	0.41	1.05	3.56	-	-	0.8047	-
Gd_5_Si_2_Ge_1.9_Ga_0.1_	[23]	0.34	1.17	4.44	-	-	0.884	-
Gd_5_Si_2_Ge_1.9_Al_0.1_	[23]	0.38	1.08	3.84	-	-	0.8403	-
Mean-field	[25]	0.5	1	3	-	-	2/3	-
3D-Heisenberg	[25]	0.365	1.386	4.797	-	-	0.8802	-
3D-Ising	[25]	0.325	1.24	4.82	-	-	0.9001	-
Tricritical mean-field	[26]	0.25	1	5	-	-	0.9333	-
